# The plasma peptidome

**DOI:** 10.1186/s12014-018-9211-3

**Published:** 2018-12-01

**Authors:** Jaimie Dufresne, Pete Bowden, Thanusi Thavarajah, Angelique Florentinus-Mefailoski, Zhuo Zhen Chen, Monika Tucholska, Tenzin Norzin, Margaret Truc Ho, Morla Phan, Nargiz Mohamed, Amir Ravandi, Eric Stanton, Arthur S. Slutsky, Claudia C. dos Santos, Alexander Romaschin, John C. Marshall, Christina Addison, Shawn Malone, Daren Heyland, Philip Scheltens, Joep Killestein, Charlotte Teunissen, Eleftherios P. Diamandis, K. W. M. Siu, John G. Marshall

**Affiliations:** 1 Ryerson Analytical Biochemistry Laboratory (RABL), Department of Chemistry and Biology, Faculty of Science, Ryerson University, 350 Victoria St, Toronto, ON Canada; 2 Institute of Cardiovascular Sciences, St Boniface Hospital Research Center, University of Manitoba, Winnipeg, Canada; 3 Division of Cardiology, Department of Medicine, McMaster University, Hamilton, Canada; 4 St. Michael’s Hospital, Keenan Chair in Medicine, University of Toronto, Toronto, Canada; 5 St. Michael’s Hospital, Keenan Research Centre for Biomedical Science, Toronto, Canada; 6 Program for Cancer Therapeutics, Ottawa Hospital Research Institute, Ottawa, Canada; 7 Clinical Evaluation Research Unit, Kingston General Hospital, Kingston, Canada; 8 Alzheimer Center, Department of Neurology, Amsterdam University Medical Centers, Vrije Universiteit, Amsterdam Neuroscience, Amsterdam, The Netherlands; 9 MS Center, Department of Neurology, Amsterdam University Medical Centers, Vrije Universiteit, Amsterdam Neuroscience, Amsterdam, The Netherlands; 10 Neurochemistry Lab and Biobank, Department of Clinical Chemistry, Amsterdam University Medical Centers, Vrije Universiteit, Amsterdam Neuroscience, Amsterdam, The Netherlands; 11 Mount Sinai Hospital Research Institute, University of Toronto, Toronto, Canada; 12 University of Windsor, Windsor, Canada; 13 International Biobank of Luxembourg (IBBL), Luxembourg Institute of Health (formerly CRP Sante Luxembourg), Strassen, Luxembourg

**Keywords:** Endogenous tryptic peptides phospho peptides, Human EDTA plasma, Organic extraction, Nano chromatography, Electrospray ionization tandem mass spectrometry, LC–ESI–MS/MS, Linear quadrupole ion trap

## Abstract

**Background:**

It may be possible to discover new diagnostic or therapeutic peptides or proteins from blood plasma using LC–ESI–MS/MS to identify, with a linear quadrupole ion trap to identify, quantify and compare the statistical distributions of peptides cleaved ex vivo from plasma samples from different clinical populations.

**Methods:**

A systematic method for the organic fractionation of plasma peptides was applied to identify and quantify the endogenous tryptic peptides from human plasma from multiple institutions by C18 HPLC followed nano electrospray ionization and tandem mass spectrometry (LC–ESI–MS/MS) with a linear quadrupole ion trap. The endogenous tryptic peptides, or tryptic phospho peptides (i.e. without exogenous digestion), were extracted in a mixture of organic solvent and water, dried and collected by preparative C18. The tryptic peptides from 6 institutions with 12 different disease and normal EDTA plasma populations, alongside ice cold controls for pre-analytical variation, were characterized by mass spectrometry. Each patient plasma was precipitated in 90% acetonitrile and the endogenous tryptic peptides extracted by a stepwise gradient of increasing water and then formic acid resulting in 10 sub-fractions. The fractionated peptides were manually collected over preparative C18 and injected for 1508 LC–ESI–MS/MS experiments analyzed in SQL Server R.

**Results:**

Peptides that were cleaved in human plasma by a tryptic activity ex vivo provided convenient and sensitive access to most human proteins in plasma that show differences in the frequency or intensity of proteins observed across populations that may have clinical significance. Combination of step wise organic extraction of 200 μL of plasma with nano electrospray resulted in the confident identification and quantification ~ 14,000 gene symbols by X!TANDEM that is the largest number of blood proteins identified to date and shows that you can monitor the ex vivo proteolysis of most human proteins, including interleukins, from blood. A total of 15,968,550 MS/MS spectra ≥ E4 intensity counts were correlated by the SEQUEST and X!TANDEM algorithms to a federated library of 157,478 protein sequences that were filtered for best charge state (2+ or 3+) and peptide sequence in SQL Server resulting in 1,916,672 distinct best-fit peptide correlations for analysis with the R statistical system. SEQUEST identified some 140,054 protein accessions, or some ~ 26,000 gene symbols, proteins or loci, with at least 5 independent correlations. The X!TANDEM algorithm made at least 5 best fit correlations to more than 14,000 protein gene symbols with p-values and FDR corrected q-values of ~ 0.001 or less. Log_10_ peptide intensity values showed a Gaussian distribution from E8 to E4 arbitrary counts by quantile plot, and significant variation in average precursor intensity across the disease and controls treatments by ANOVA with means compared by the Tukey–Kramer test. STRING analysis of the top 2000 gene symbols showed a tight association of cellular proteins that were apparently present in the plasma as protein complexes with related cellular components, molecular functions and biological processes.

**Conclusions:**

The random and independent sampling of pre-fractionated blood peptides by LC-ESI-MS/MS with SQL Server-R analysis revealed the largest plasma proteome to date and was a practical method to quantify and compare the frequency or log_10_ intensity of individual proteins cleaved ex vivo across populations of plasma samples from multiple clinical locations to discover treatment-specific variation using classical statistics suitable for clinical science. It was possible to identify and quantify nearly all human proteins from EDTA plasma and compare the results of thousands of LC–ESI–MS/MS experiments from multiple clinical populations using standard database methods in SQL Server and classical statistical strategies in the R data analysis system.

**Electronic supplementary material:**

The online version of this article (10.1186/s12014-018-9211-3) contains supplementary material, which is available to authorized users.

## Introduction

In theory all tissues and cells are in constant communication via endo/exocytosis or secretion with the extracellular space that is directly or indirectly contiguous with the circulatory systems and thus the blood fluids [1, 2]. Tryptic peptides analyzed by high pressure liquid chromatography (HPLC) [3] with electrospray ionization [4] followed by ion trap MS/MS fragmentation (LC–ESI–MS/MS) have been shown to reliably identify and quantify peptides from Eukaryotic samples [5–7]. Only a few hundred blood proteins may be detected by preparative 1D poly acrylamide gel electrophoresis (PAGE) [8] or analytical 2D PAGE [9]. A direct comparison of electrophoresis followed by LC–ESI–MS/MS compared to partition chromatography followed by LC–ESI–MS/MS showed that direct chromatographic methods had much greater sensitivity [8]. Plasma or serum proteins may be digested with trypsin and identified by fitting the MS/MS spectra to non-tryptic peptides [10–13]. Exogenous tryptic digestion of blood fluids results in the highly redundant analysis of albumin, apolipoproteins, immunoglobulins [14, 15], and other well-known blood proteins [1, 2]. In contrast, examination of endogenous peptides shows a greater representation of apparently cellular proteins [16, 17]. The agreement on the identified proteins of human blood fluids from MS/MS spectra between “Fully Tryptic” peptides that are constrained to end in R or K [8, 15] versus the “No Enzyme” peptides that are free to end with any of the 20 amino acids [10, 11] is powerful evidence for the veracity of LC–ESI–MS/MS of tryptic peptides [14, 18, 19]. The statistical sufficiency of MS/MS correlation with an ion trap has been confirmed by independent experimental methods including computing MS/MS to peptide p-values (and FDR q-values), or comparison to noise MS/MS and random MS/MS simulations [20, 21] and is in agreement with the results from 300,000 synthetic test peptides [22] or pure viral cultures [23]. Blood fluid contains a weak tryptic activity [24] that apparently may cleave endogenous peptides in vivo (peptidome) but endogenous proteolytic activities may also generate high levels of some of these same peptides ex vivo (degradome) [25, 26] since these two pools show some overlap [27]. Incubation of plasma at room temperature seemed to result in an apparent steady state where peptides are being created by endoproteinases and degraded by exopeptidases [27–29]. To date the isolation and identification of the endogenous peptides from human plasma has seemed technically challenging [30]. The most abundant endogenous peptides of human blood fluid were first identified by C18 partition chromatography followed by MALDI-MS/MS with a Qq-TOF or LC–ESI–MS/MS with an ion trap, tandem mass spectrometer [8, 28]. The problem of low signal strength from blood peptides was first addressed by examining haemofiltrate [31] and/or the use of sensitive MALDI analysis [32, 33]. The use of precipitation and selective extraction of the protein pellet was shown to be superior to precipitation and analysis of the ACN supernatant [34], ultra-filtration, [11] albumin depletion chromatography [35], partition chromatography (DEAE ion exchange & C18) or C18 alone [8]. Organic extraction may have some advantage to detect cellular proteins compared to the redundant identification of canonical circulating proteins frequently observed from depletion chromatography [10], ultrafiltration [11], or partition chromatography [8, 15] of blood proteins followed by trypsin digestion. Precipitating the blood peptides for organic/water extraction has resulted in the identification of cellular proteins and regulatory molecules growth factors [16, 17, 36, 37]. It will be necessary to identify, quantify and compute the statistical distributions of the endogenous tryptic peptides cleaved from the proteins ex vivo in blood plasma compared to ice cold controls to understand and compare treatment versus pre-analytical variation in different clinical populations and controls.

Precipitation of the sample with nine volumes of acetonitrile results in a pellet that contains almost all the peptides and proteins that may be selectively extracted from the insoluble pellet by a stepwise gradient of acetonitrile/water for collection over C18 followed by LC–ESI–MS/MS that provides high signal-to-noise ratios [38] and combined with nanospray resulted in the identification of cellular proteins and regulatory molecules such as interleukins and growth factors. Here the proteins in blood that were cleaved in human plasma ex vivo provided convenient and sensitive access to almost all human proteins and classical statistical approaches detected variation between clinical populations, female samples and ice cold degradation controls.

## Materials and methods

### Materials

The HPLC was an Agilent 1100 (Santa Clara CA USA). The linear ion trap mass spectrometer was a LTQ XL (Thermo Electron Corporation, Waltham, MA, USA). Anonymous human EDTA plasma with no identifying information was obtained from the multiple clinical locations of St Joseph’s Hospital of McMaster University, the Ontario Tumor Bank of the Ontario Institute of Cancer Research, St Michaels Hospital Toronto, Amsterdam University Medical Centers, Vrije Universiteit Amsterdam, and IBBL Luxembourg under Ryerson Ethic Review Board Protocol REB 2015-207. The arbitrarily-selected disease population samples were from patients that received a confirmed diagnoses of the disease indicated at the source institution. The plasma samples were collected before therapeutic intervention and no additional information about the samples were made available. C18 ZipTips were obtained from Millipore (Bedford, MA). C18 HPLC resin was from Agilent (Zorbax 300 SB-C18 5-micron). Solvents were obtained from Caledon Laboratories (Georgetown, Ontario, Canada). All other salts and reagents were obtained from Sigma-Aldrich-Fluka (St Louis, MO) except where indicated.

### Sample preparation

The samples stored at − 80 °C were thawed on ice and briefly vortexed before pipetting 200 μL in the bottom of a 2 ml sample tube on ice. The endogenous tryptic peptides or tryptic phospho peptides (i.e. without exogenous digestion) were extracted in a step gradient of organic solvents, dried and collected by preparative C18 [38]. Disposable plastic 2.0 ml sample tubes and plastic pipette tips were used to handle samples. The 200 μl of EDTA plasma samples were precipitated with 90% acetonitrile [34], that contains few peptides, followed by the selective extraction of the pellet using a step gradient to achieve selectivity across sub-fractions and thus greater sensitivity [38]. Human EDTA plasma samples (200 μl) were precipitated with 9 volumes (1800 μL) of 100% acetonitrile (final 90% v/v). First, the acetonitrile suspension was separated with a centrifuge at 14,000 RCF for 5 min. Next, the acetonitrile supernatant was collected, transferred to a fresh sample tube and freeze dried in a rotary lyophilizer. The organic precipitate (pellet) that contains a much larger total amount of endogenous polypeptides [17, 36] was manually re-suspended in a 200 μL volume using a step gradient of increasing water content to yield 10 fractions from the most organic soluble 90% ACN supernatant to 10% ACN, followed by 100% H_2_O, and then 5% formic acid [38]. At each step the extract was clarified with a centrifuge at 14,000 RCF for 5 min. Supernatant from each step fraction was dried under vacuum in a rotary lyophillizer and stored at − 80 °C for analysis.

### Preparative micro C18 chromatography

Extracted peptides of EDTA plasma were then re-dissolved in 5% formic acid for preparative C18 chromatography (0.5 μL ZipTip). Solid phase extraction with C18 for LC–ESI–MS/MS were performed as previously described [8, 15, 28, 36, 37]. The C18 chromatography resin (zip tip) was wet with 65% acetonitrile before equilibration in water with 5% acetonitrile and 5% formic acid. The plasma extract was dissolved in 200 μL of 5% formic acid in water. The resin was washed with at least five volumes of the same binding buffer. The resin was eluted with 2 μL of 65% acetonitrile in 5% formic acid. The preparative resin was discarded after a single use.

### LC–ESI–MS/MS

Disease and matched normal control sample fraction sets were replicated in blocks over each of five LTQ XL Linear Quadrupole ion traps that were cleaned and tested between patients. The linear quadrupole ion traps were tested for sensitivity by infusion with Glu Fib and angiotensin II. To entirely prevent any possibility of cross contamination between patient step-fraction sets, a new analytical column and emitter tip was fabricated for each patient. Sensitivity and accuracy of the LC–ESI–MS/MS was tested using a mixture of cytochrome c, glycogen phosphorylase B and alcohol dehydrogenase [20, 21, 27, 39, 40] prior to recording the peptides from each patient sub-fraction set. Stepwise extractions were collected and desalted over C18 preparative micro columns, eluted in 2 μL of 65% ACN and 5% formic acid, and then were diluted tenfold with 0.1% formic acid in water before loading into a 20 μL metal sample loop with manual injection onto the analytical column via a Rhodynne injector. Endogenous peptide samples were analyzed over a discontinuous gradient generated at a flow rate of ~ 10 microlitres per minute split upstream of the injector during recording to about ~ 200 nl per minute. Separation was performed with a C18 (150 mm × 0.15 mm) fritted capillary column. Acetonitrile profile was at 5% during injection, was ramped to 12% after 5 min and then increased to 65% over ~ 60 min, remained at 65% for 5 min, decreased to 50% for 15 min and then declined to a final proportion of 5% prior to injection of the next step fraction from the same patient. Nano HPLC effluent was analyzed by nanospray ionization with detection by MS and fragmentation by MS/MS with a linear quadrupole ion trap [41]. The instrument was set to collect the precursor for up to 200 milli seconds prior to MS/MS fragmentation with up to four fragmentations per precursor ion that were combined. On average, about 10 independent patient plasma samples from each of 13 distinct sample sets with control versus disease treatments from multiple institutions or studies (McMaster, St Michael’s Hospital, Hospital Zentraal, Ontario Tumor Bank, and IBBL) were precipitated, fractionated over a step gradient and collected over C18 for manual injection. In the case of heart attack where low variation was observed, greater sampling density was employed.

### Sampling and analysis strategy

Endogenous tryptic peptides extracted from the disease and/or matched control treatments (ovarian cancer, breast cancer, sepsis, Alzheimer’s dementia, multiple sclerosis and heart attack) and ice cold plasma controls were randomly and independently sampled by LC–ESI–MS/MS [27] (13 treatments with 1508 successful LC–ESI–MS/MS experiments). Independent patient samples from the each of the disease and normal treatments were separated into 10 sub-fractions that were randomly and independently sampled by the linear quadrupole ion trap that provided the precursor ion m/z and intensity values. Disease and matching control sample fraction sets were replicated in blocks over each of five identical LTQ XL Linear Quadrupole ion traps. Accession numbers, actual and estimated masses [M + H]^+^, correlated peptide sequences, peptide and protein scores, resulting protein sequences and other associated data were captured and assembled together in an SQL Server relational database for analysis with the R generic statistical analysis system [18].

#### Correlation analysis

Correlation analysis of ion trap data was performed with X!TANDEM [42] and SEQUEST [6] algorithms to match tandem mass spectra to peptide sequences from the Homo sapiens RIKEN, IMAGE, RefSeq, ENSEMBL, UNIPROT, UNIPARC and SwissProt Federated Library of 157,478 protein sequences that differ by at least one amino acid. Correlation algorithms may match one MS/MS spectra to more than one peptide sequence, or charge state, or to the same peptide found in many proteins, that can be filtered using SQL Server database system to avoid redundant correlations and over interpretation of the data [15, 27, 39, 40, 43]. Endogenous peptides were searched as fully tryptic peptides on separate servers for the SEQUEST and X!TANDEM algorithms and these results were combined in an SQL Server relational database. The ion trap data was analyzed within ± 3 m/z from fully tryptic precursor peptides considered from 300 to 2000 m/z with a tolerance of 0.5 Da error in the fragments with up to three missed cleavages [42]. The entire correlation procedure for fully tryptic peptides was repeated with the additional consideration of phosphate on serine, threonine or tyrosine residues as specified in the X!TANDEM and SEQUEST software algorithms: The precursor intensity and frequency counts from the 13 plasma treatments × 2 independent correlations (tryptic and phospho tryptic) resulted in 26 treatments for statistical analysis.

#### Data sorting, transformation and visualization

The peptide identity and [M + H]^+^ were computed from the MS/MS spectra by the SEQUEST and X!TANDEM algorithms. The X!TANDEM and SEQUEST correlation algorithms can automatically match one MS/MS spectra to more than one peptide sequence or charge state that may be subsequently filtered out using the SQL Server database system to avoid redundant correlations [15, 27, 39, 40, 43]. Only the single best fit (Rank 1) peptide from the MS/MS at charge states of + 2 versus + 3 were accepted with additional acetylation or oxidation of methionine and with possible loss of water or ammonia. The results from the LC–ESI–MS/MS spectra together with the results of the correlation algorithms were parsed into an SQL Server that was analyzed by the open source R statistical analysis system [14, 18, 39, 40, 44]. A continuous variable (such as log_10_ intensity) that is randomly and independently sampled across multiple clinical sites and treatments, and that is linear and Gaussian by quantile plot, is an ideal candidate for means comparison by ANOVA followed by Tukey–Kramer Honestly significant differences test, a classical statistical analysis used in clinical research, basic research, engineering and agricultural science [27, 28, 39, 40, 44, 45]. The charts, tables and statistical tests for the 1.9 million filtered data points were created using the library Rcmdr in R. The MS/MS correlations p-values and intensity values of the fully tryptic and/or phospho tryptic peptides were computed per gene symbol using the SQL SERVER/R data system. The intensity data was log_10_ transformed, tested for normality and analyzed by means, standard errors and ANOVA with the R statistical analysis system.

## Results

The number of sample fractions that showed intense LC–ESI–MS/MS spectra > E4 counts seemed to vary between treatments and it was more difficult to detect peptides from control plasma collected on ice compared to the clinical samples (Table 1). Fully tryptic peptides and/or phospho peptides from ~ 14,000 gene symbols (X!TANDEM) or nearly all 26,000 gene symbols (SEQUEST) of the known human proteins were observed by a progressive stepwise extraction of the organic protein pellet of human EDTA plasma. The stepwise extraction apparently revealed peptides from many cellular proteins and regulatory proteins such as growth factors, cytokines, chemokines, necrosis factors and interleukins in circulation and provided a comprehensive analysis of the endogenous tryptic peptides in human plasma. (Additional file 1: Table S1; Additional file 2: Table S2). The analysis of the endogenous peptides by nano-electrospray ionization with a linear quadrupole ion trap showed highly significant protein p-values and FDR-corrected q-values from the best fit of MS/MS spectra to tryptic peptides.

**Table 1:** The number of mgf files with MS/MS spectra > E4 counts search against the human proteins by X!TANDEM and SEQUEST

Treatment ID#	MGF file count	TreatmentName
1	115	Alzheimer control
2	115	AlzHeimer control STYP
3	120	AlzHeimer
4	120	Alzheimer STYP
5	89	Cancer breast
6	89	Cancer breast STYP
7	50	Cancer control
8	50	Cancer control STYP
9	90	Cancer ovarian
10	90	Cancer ovarian STYP
11	12	Ice cold control
12	12	Ice cold control STYP
13	211	Heart attack arterial
14	211	Heart attack arterial STYP
15	267	Heart attack venous control
16	267	Heart attack venous control STYP
17	121	Heart attack venous
18	121	Heart attack venous STYP
19	121	Multiple sclerosis control
20	121	Multiple sclerosis control STYP
21	122	Multiple sclerosis
22	122	Multiple sclerosis STYP
23	100	Sepsis
24	100	Sepsis STYP
25	90	Sepsis control
26	90	Sepsis control STYP
LCMS runs searched	1508	Fully Tryptic
LCMS runs searched	1508	Phospho Tryptic STYP

A total of 1508 mgf files with high intensity spectra greater than E4 counts were searched both as fully tryptic and phospho tryptic peptides and the results compared as separate treatments

### LC–ESI–MS/MS

The pool of endogenous tryptic peptides (TRYP) and/or phosphopeptides (TRYP-STYP) were extracted in organic solvent/water for collection over C18 [46, 47] and analyzed by random and independent sampling from independently replicated disease and normal samples without replacement by analytical C18 LC–ESI–MS/MS (Table 1). The MS/MS spectra were correlated to the tryptic peptides of the federated library of human proteins by the SEQUEST and X!TANDEM algorithms. Some 15,043,678 MS/MS spectra from precursor ions of typically greater than 10,000 arbitrary intensity counts from 1508 nano spray LC–ESI–MS/MS runs were recorded (Table 2).

**Table 2:** The filtering of proteins from endogenous tryptic peptides (TRYP) or tryptic phospho peptides (STYP) from the stepwise extraction of human EDTA plasma with a mixture of organic solvent and water where MS/MS correlations from precursor ions of greater than ~ E4 (10,000) arbitrary counts were accepted from 1508 LC–ESI–MS/MS runs

MS/MS spectra	15,968,550
*Protein filtering*	
Protein library	157,478
Correlations	94,483,230
Best charge state (Filter 1)	
Protein accessions	156,280
Correlations	19,889,758
Best charge state and peptide sequence (Filter 2)	
Protein accessions	156,279
Correlations	19,197,152
*Rank 1 Peptides*	
Peptide identification count	19,197,152
Distinct peptide identification count	4,631,474
Distinct peptide sequence count	1,916,672
Peptides from ≥ 3 correlations per protein	486,895

About 12% of MS/MS spectra from precursors greater than E4 counts were accepted as the best fit to a fully tryptic peptide or phosphopeptide

### SQL analysis of MS/MS spectra to peptides and proteins

A total of 94,483,230 redundant MS/MS spectra to peptide matches (precursor intensity ≥ E4 counts) to the library of 157,478 proteins from a total of 19,197,152 correlations to 156, 279 different protein accessions collapsed in the SQL Server to 1,916,672 distinct, Rank 1 correlations to 26,251 possible gene symbols (Table 2).

### Identification of peptides by SEQUEST and X!TANDEM

**Fig. 1:**
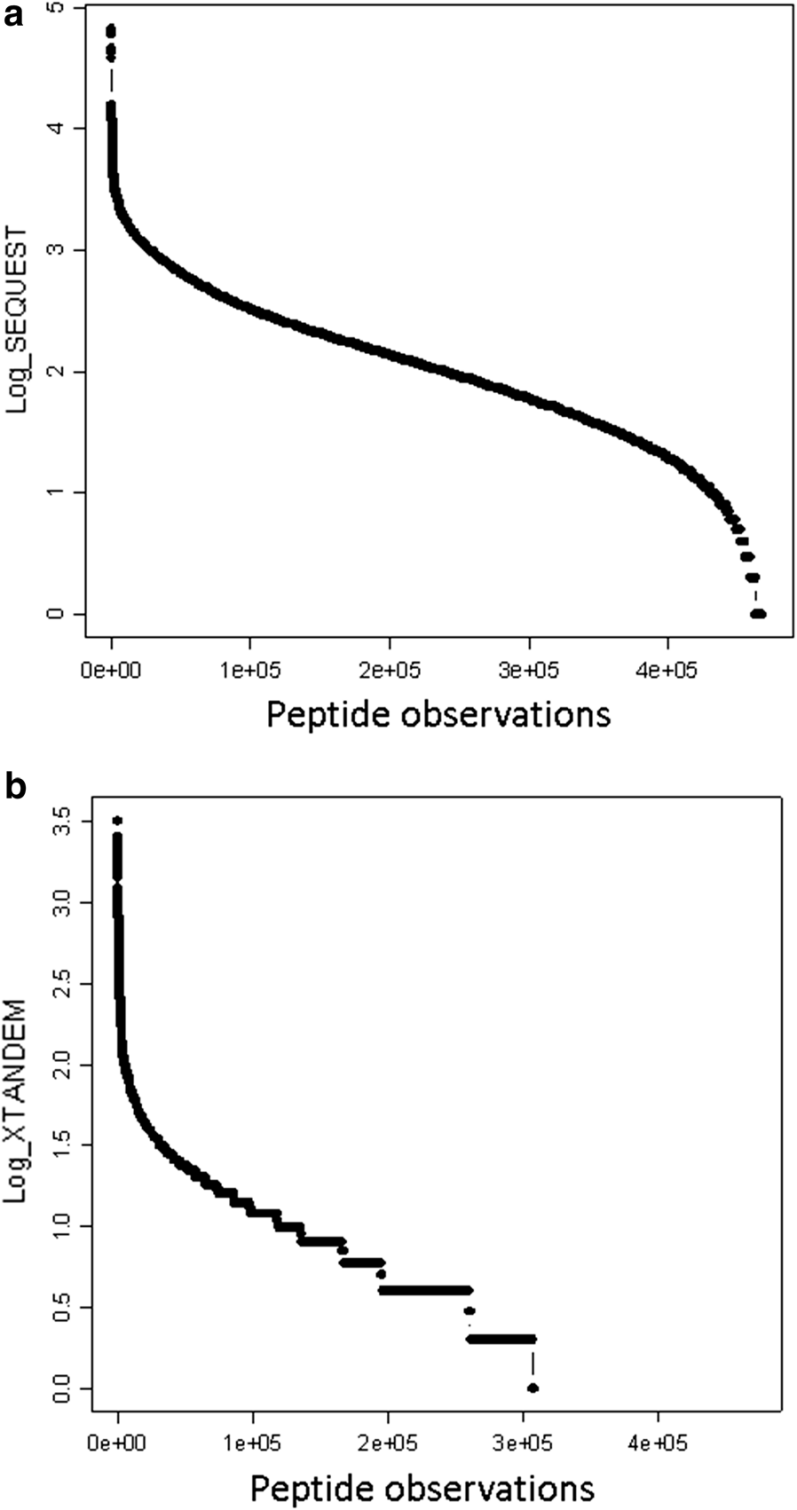
The human endogenous tryptic peptides and/or phosphopeptides where precursor intensity is greater than E4 (10,000) arbitrary detector counts after selecting the best fit of MS/MS spectra from 2+ or 3+ ions and selecting the single best peptide fit for each MS/MS fragmentation spectrum (Filter 2). **a** the SEQUEST algorithm; **b** the X!TANDEM algorithm

**Fig. 2:**
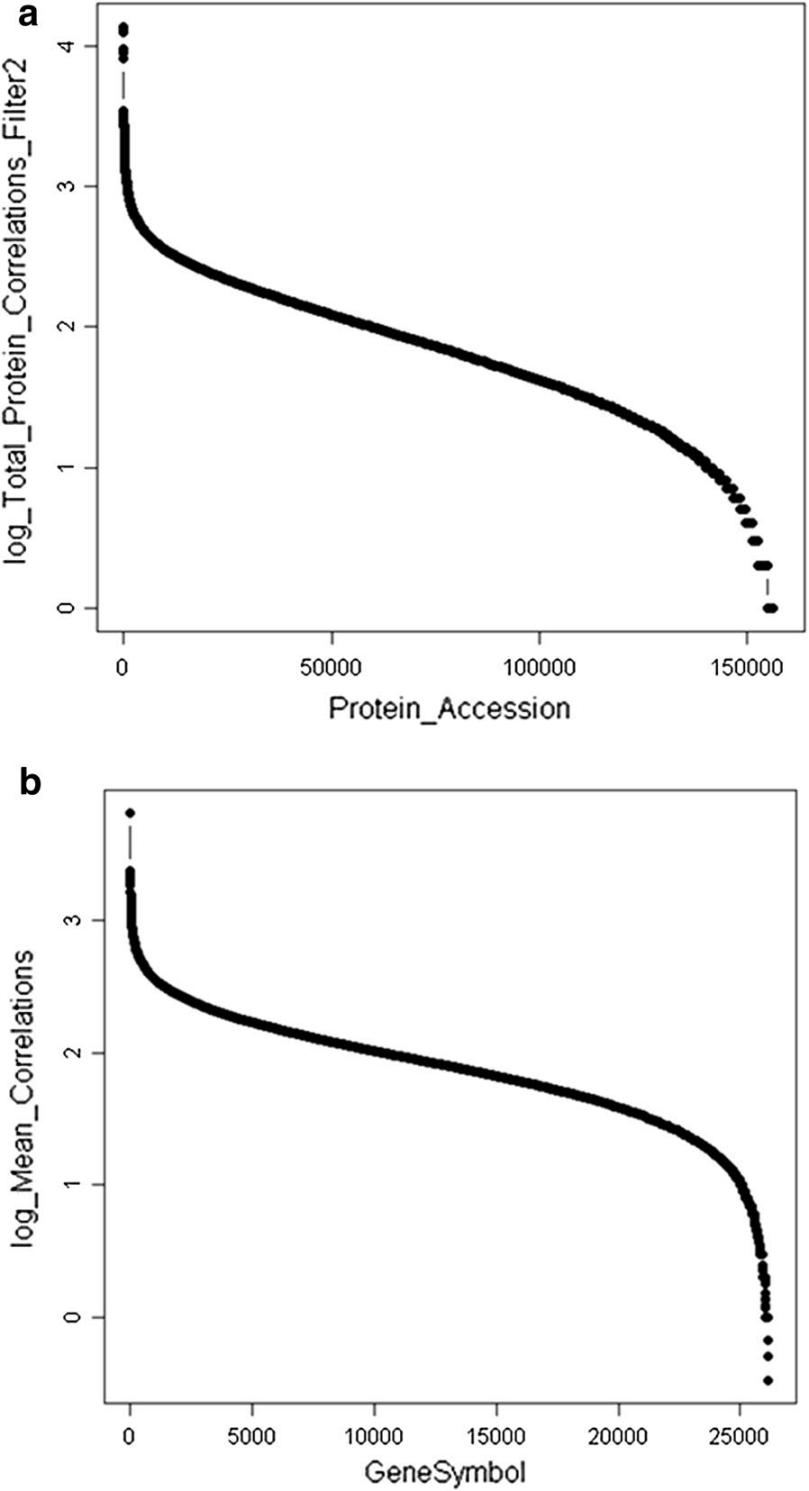
The human protein gene symbols identified by endogenous tryptic peptides and/or phosphopeptides using Filter 2 where precursor intensity is greater than E4 (10,000) arbitrary detector counts from the SEQUEST plus X!TANDEM algorithm after selecting the best fit of MS/MS spectra from 2+ or 3+ ions and selecting the single best peptide fit for each MS/MS fragmentation set and thus rejecting the redundant correlation of the same MS/MS spectra more than once. **a** log peptide counts per protein accession; **b** log_10_ peptide counts per gene symbol

The SEQUEST algorithm was very sensitive and tryptic peptides and/or phosphopeptides from immunoglobulin molecules were detected as many as ten thousand times with some 4 * E5 peptides correlated at least 3 times (Fig. 1a). The SEQUEST results showed a bias toward identifying peptides from large proteins such as titin, nebulin, spectrin, obscurin, microtubule cross linking factor, ANAK, and others that result from noise or miss-correlation [20, 21, 48]. In contrast, X!TANDEM detected about 1.5 * E5 tryptic and phospho peptides at least 3 times (Fig. 1b). Combining the results of SEQUEST and X!TANDEM showed a total of about 140,000 protein accessions (Fig. 2a) and about 24,000 gene symbols (Fig. 2b) that were identified at least 5 times. Thus nearly every human gene symbol was provisionally identified by LC–ESI–MS/MS from organic extraction of plasma by the heuristic SEQUEST algorithm that does not provide a direct measure of confidence with respect to random expectation.

### Statistical distributions of individual peptides from X!TANDEM

**Fig. 3:**
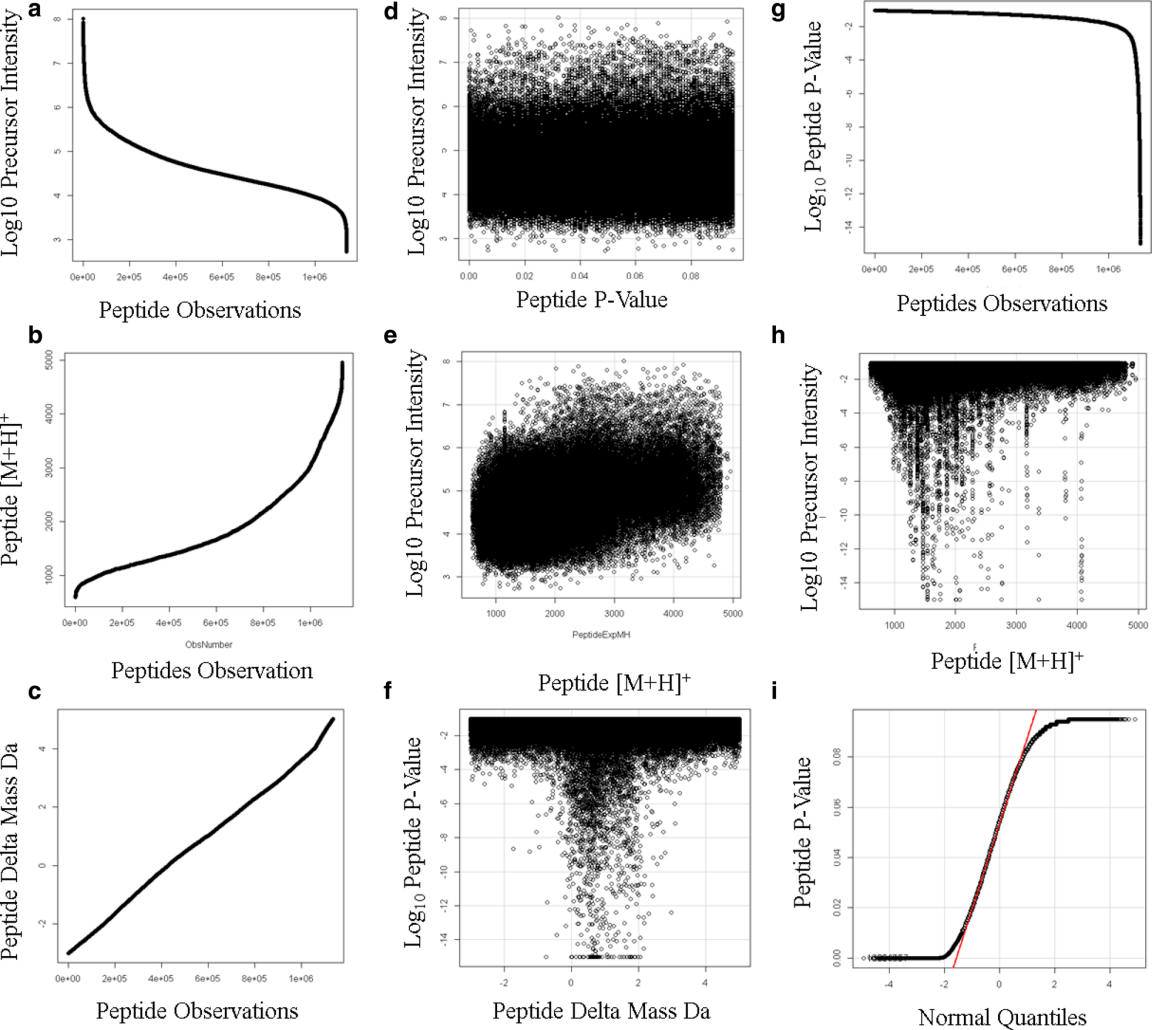
The distributions of the endogenous Rank 1 tryptic peptides correlated by the X!TANDEM algorithm from human EDTA plasma. **a** The sorted log10 precursor intensity values; **b** the sorted peptide [M + H]^+^ values; **c** the sorted peptide delta mass values; **d** the scatter plot log10 peptide p-values versus precursor intensity; **e** log10 intensity versus peptide [M + H]^+^ Residual standard error: 0.5488 on 1135580 degrees of freedom Multiple R-squared: 0.2236, Adjusted R-squared: 0.2236 F-statistic: 3.27e + 05 on 1 and 1,135,580 DF, p-value: < 2.2e−16); **f** log10 peptide p-values versus the delta mass value; G, sorted log10 peptide p-value; **h** log10 peptide p-value versus [M + H]^+^; **i** quantile plot of peptide p-values

The X!TANDEM algorithm generates a *p-*value that the experimental MS/MS matched the predicted spectra of a peptides in the human protein library and so it was possible to compute the probability distributions for peptides, protein accessions and gene symbols. X!TANDEM matched 1,135,806 MS/MS spectra to peptide sequences (Fig. 3). The peptide intensity values ranged from E8 to E4 counts that is over 4 orders of magnitude, approximated a normal distribution, and the variation in precursor intensity values was well-explained over peptides by ANOVA (p ≤ E-15) (Fig. 3a). The [M + H]^+^ values ranged from about 900 to 5000 Da, and approximated a normal distribution (Fig. 3b). The peptide delta mass values were linear from from − 2 to + 4 Da and showed a close fit to a Gaussian distribution (Fig. 3c). The precursor intensity had little effect on the peptide p-values that ranged from E-1 to E-15 (Fig. 3d). The scatter plot of peptide intensity versus [M + H]^+^ showed an increasing trend (p-value: ≤ 2.2e−16) consistent with greater momentum on detector impact (Fig. 3e). There was a Gaussian relationship between Log p-values and delta mass (Fig. 3f). The individual peptide p-values ranged from E-1 to E-14 (Fig. 3g). The most significant peptide p-values ranged from 1000 to 4000 Da (Fig. 3h). The peptide p-values did not closely approximate a normal or Gaussian curve in the outer quantiles (Fig. 3i).

#### Computation of X!TANDEM results per protein accession

**Fig. 4:**
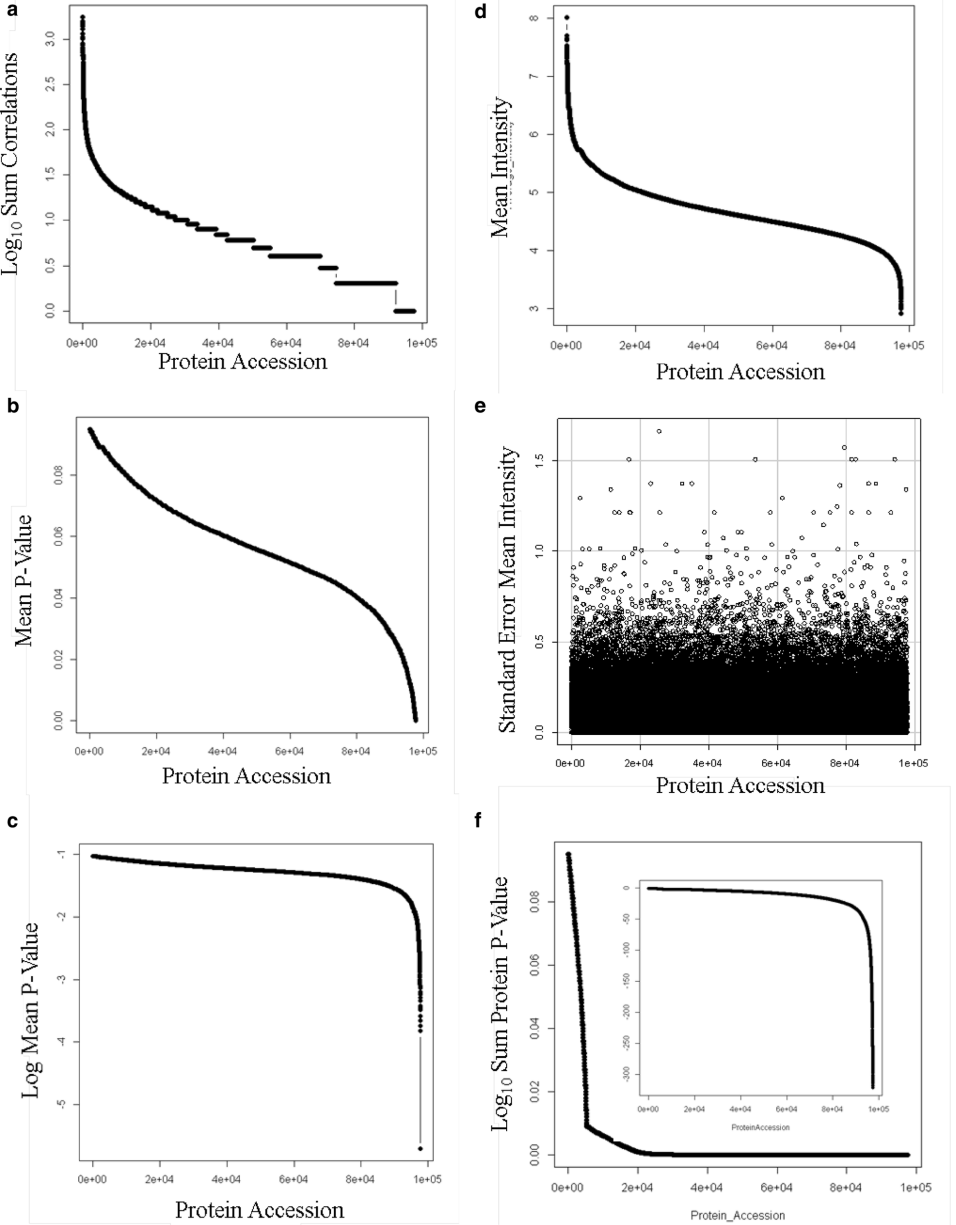
The distributions of the endogenous Rank 1 tryptic peptides correlated by the X!TANDEM algorithm from human EDTA plasma at the level of protein. **a** The peptide to protein accession count; **b** the average peptide p-value per protein accession; **c** the log10 average peptide p-value per protein accession; **d** log10 precursor intensity value per protein accession; **e** the standard error of the protein accession log10 intensity; **f** the cumulative p-value per protein (inset cumulative log10 p-value per protein accession)

The X!TANDEM algorithm computed at least three correlations to ~40,000 protein accessions (Fig. 4a). The average p-values of the observed peptide sequences ranged from E-1 to E-5 (Fig. 4b, c). The average intensity values significantly varied over protein accessions (ANOVA p ≤ E-15) that ranged from E8 to E4 arbitrary counts (Fig. 4d). The standard error of the peptide intensity was typically less than 0.5 log_10_ units (Fig. 4e). The cumulative p-values at the level of the protein accession that ranged from E-3 to E-300 was estimated from the average p-value and number of independent observations (Fig. 4f).

#### Computation of X!TANDEM at the level of gene symbols

**Fig. 5:**
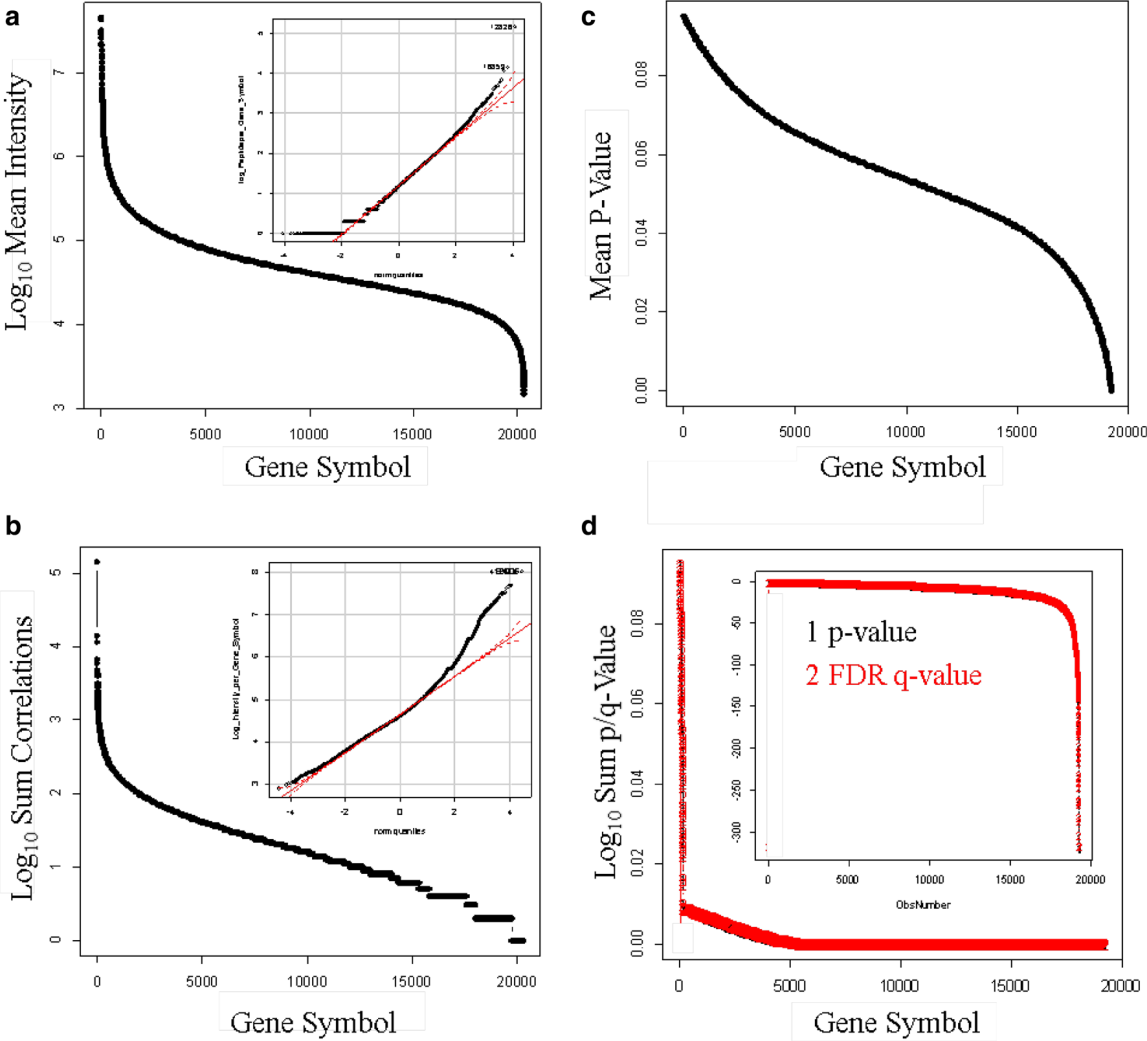
The distributions of the Rank 1 endogenous tryptic peptides correlated by the X!TANDEM algorithm from human EDTA plasma from the best protein accession per gene symbols. **a** log peptides observed per gene symbol (inset quantile plot of peptide frequency count); **b** log mean precursor intensity per gene symbol (inset quantile plot of log_10_ intesity); **c** log mean p-value per gene symbol; **d** cumulative log_10_ p-value per gene symbol (inset log_10_ cumulative p-value per gene symbol)

Genes may be mutated to create new protein sequences [49] RNA may be spliced [50, 51] and the proteins may be processed [52, 53] leading to different variant forms of related proteins that may sometimes share a similar gene symbol in SQL Server. Selecting the data from the single protein accession with the most peptide correlations per gene symbol is a simple means to summarize the protein results that avoids redundant correlations to peptides shared by homologous proteins. The results of the X!TANDEM algorithm mapped to ~ 19,000 different gene symbols, open reading frames, or loci with ~ 14,000 gene symbols that showed ≥ 5 independent peptides with greater than E4 intensity and the log_10_ peptide frequency was normally distributed (Fig. 5a). The mean log peptide intensity per gene symbol ranged over at least 3 orders of magnitude from ~ E8 to E4 arbitrary counts that approached a Gaussian distribution by quantile plot (Fig. 5b). The average MS/MS to peptide p-values per gene symbol ranged from E-1 to E-5 (Fig. 5c). Estimating the cumulative protein p-value from the average and the number of observations showed that ~ 14,000 gene symbols show a type I error (p-value) and False Discovery Rate (q-value) of 0.001 or lower (Fig. 5d).

### Analysis of intensity means and error

**Fig. 6:**
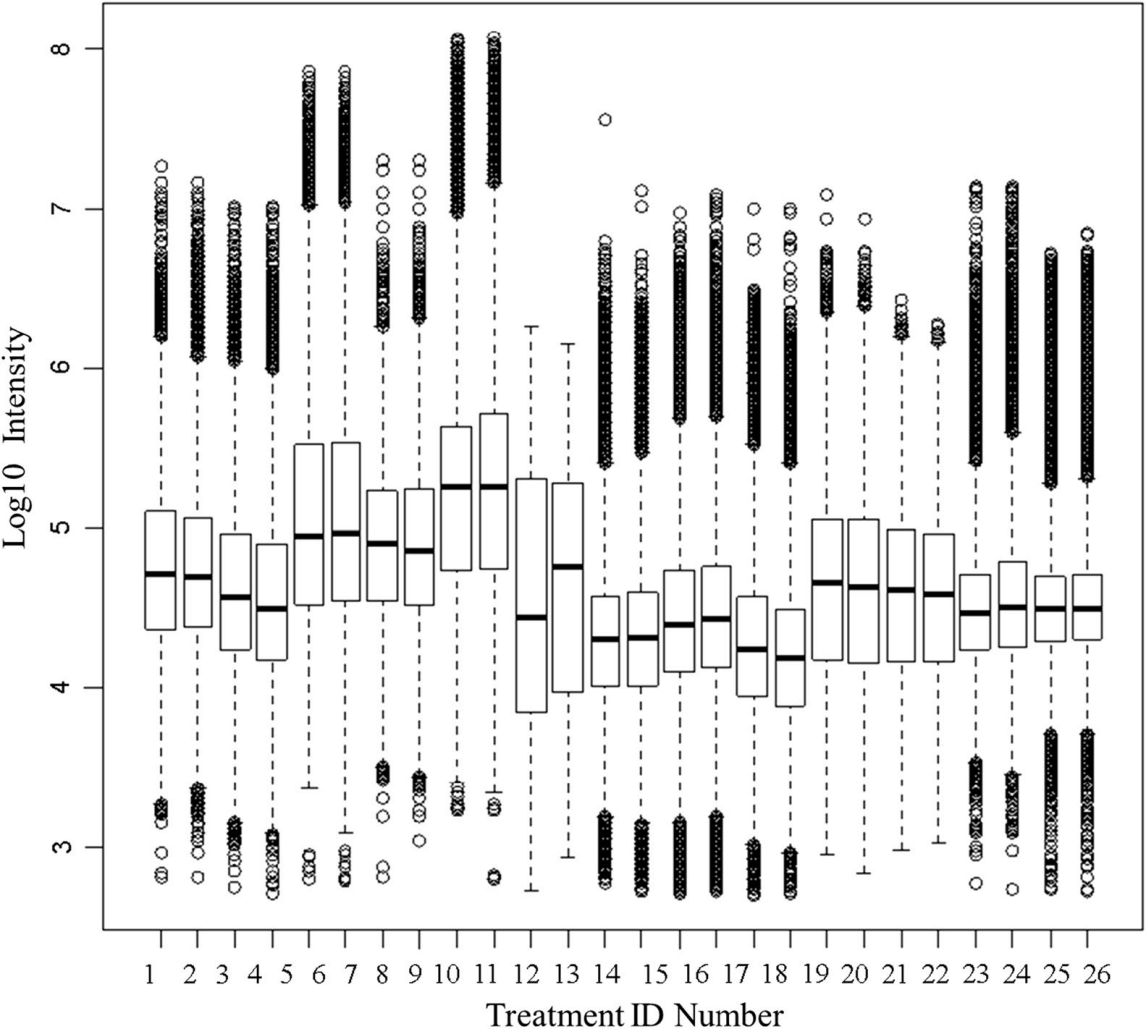
The box plot and ANOVA of log_10_ peptide intensity from 26 control and disease EDTA plasma samples. Treatment ID numbers: 1, Alzheimer normal; 2, Alzheimer normal control STYP; 3, AlzHeimer’s dementia; 4, Alzheimer’s dementia STYP; 5, Cancer_breast; 6, Cancer_breast_STYP; 7, Cancer_control; 8, Cancer_control_STYP; 9, Cancer_ovarian; 10, Cancer_ovarian_STYP; 11, Ice Cold; 12, Ice Cold STYP; 13, Heart attack Arterial; 14 Heart attack Arterial_STYP; 15, Heart attack normal control, 16, Heart attack normal Control STYP; 17, Heart attack; 18, Heart attack STYP; 19, Multiple Sclerosis normal control; 20, Multiple Sclerosis normal control STYP; Multiple Sclerosis; 22, Multiple Sclerosis STYP, 23 Sepsis; 24, Sepsis STYP; 25, Sepsis normal control; 26, Sepsis normal control STYP. The ANOVA analysis across treatments produced an F Statistic of 13,898 and a p-value of 2e−16*** (Additional file 3: Table S3). STYP: serine, threonine, tyrosine phosphorylation

**Fig. 7:**
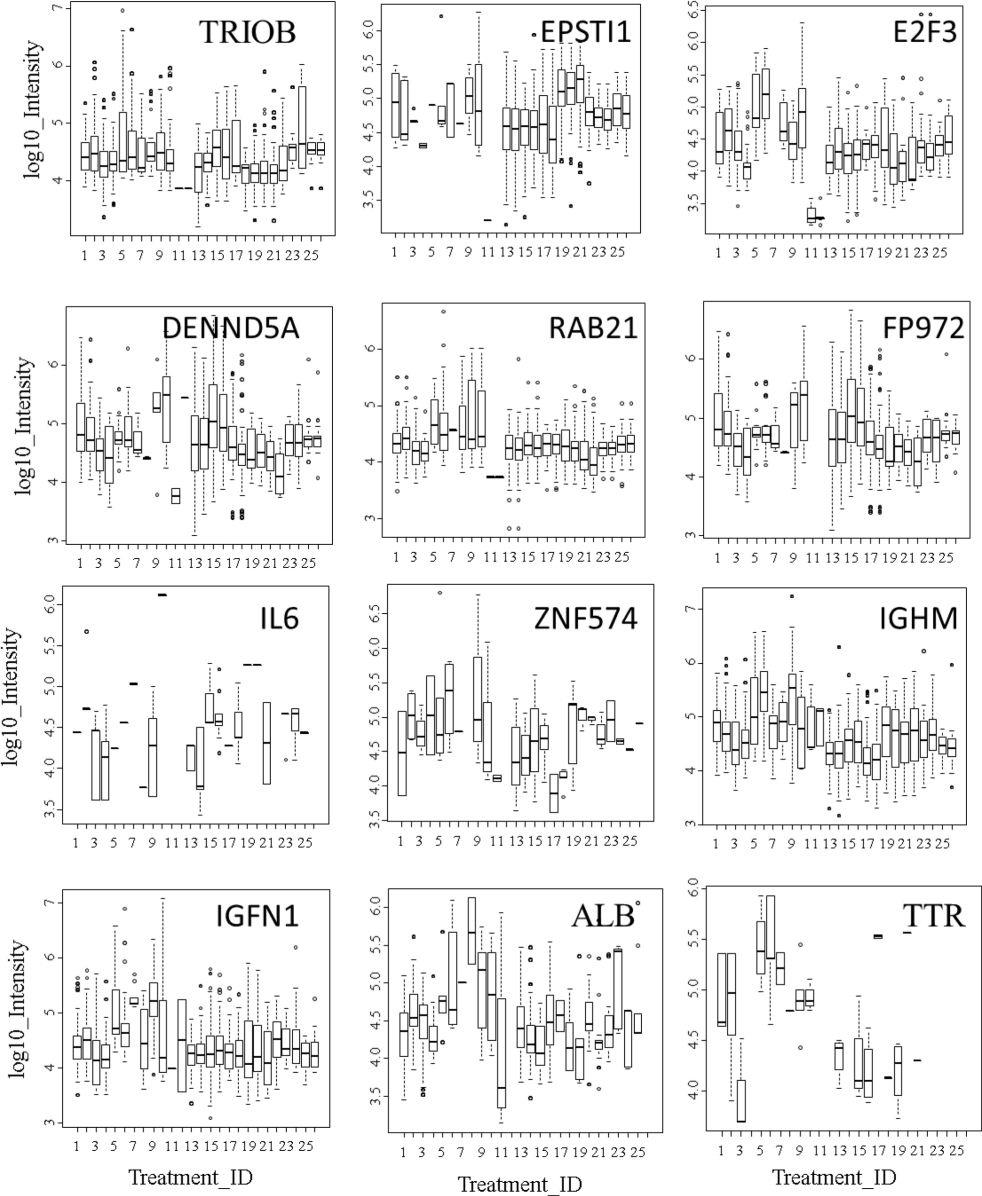
The box plot and ANOVA of log_10_ peptide intensity from 26 control and disease EDTA plasma samples for some frequently observed gene symbols. Treatment ID numbers: 1, Alzheimer normal; 2, Alzheimer normal control STYP; 3, AlzHeimer’s dementia; 4, Alzheimer’s dementia STYP; 5, Cancer_breast; 6, Cancer_breast_STYP; 7, Cancer_control; 8, Cancer_control_STYP; 9, Cancer_ovarian; 10, Cancer_ovarian_STYP; 11, Ice Cold; 12, Ice Cold STYP; 13, Heart attack Arterial; 14 Heart attack Arterial_STYP; 15, Heart attack normal control, 16, Heart attack normal Control STYP; 17, Heart attack; 18, Heart attack STYP; 19, Multiple Sclerosis normal control; 20, Multiple Sclerosis normal control STYP; Multiple Sclerosis; 22, Multiple Sclerosis STYP, 23 Sepsis; 24, Sepsis STYP; 25, Sepsis normal control; 26, Sepsis normal control STYP. The ANOVA analysis across treatments produced an F Statistic of 13,898 and a p-value of 2e−16***. STYP: serine, threonine, tyrosine phosphorylation. Note that many proteins were not detected in the ice cold plasma

The grand mean Log_10_ precursor intensity of protein gene symbols showed significant differences across the 12 clinical populations versus the ice cold plasma controls values as judged by box plots computed in R (Fig. 6). Mean log_10_ precursor intensity showed highly significant variation between the separate disease and control sample treatments by one-way ANOVA (p < 2 E-16) (Fig. 6). The results indicate that there was significant variation in the ex vivo cleavage of proteins between the different populations of clinical samples. Global differences in peptide intensity between treatments may confound subsequent comparisons of individual proteins across disease and normal control populations. However, while the grand average intensity was not greatly different between the many treatments, there were some proteins that apparently differed in observation frequency or peptide intensity between normal and disease treatments from different clinical populations versus the ice cold controls. The SQL Server and R software permits the intensity and frequency of any protein to be compared across the clinical treatments with a complete statistical analysis of the linear and Gaussian log_10_ intensity results. The box plots of cellular proteins that were frequently observed like RAB21 or DENND5A (Fig. 7) seem to indicate there is a significant variation in the log_10_ mean peptide intensity pattern of cellular proteins cleaved in the disease and matched normal samples from different clinical locations, female samples or samples on ice. For example, large differences were observed across treatments in peptides cleaved ex vivo from transferrin (TTR) and albumin (ALB) between clinical populations or the ice cold controls.

### STRING analysis

**Fig. 8:**
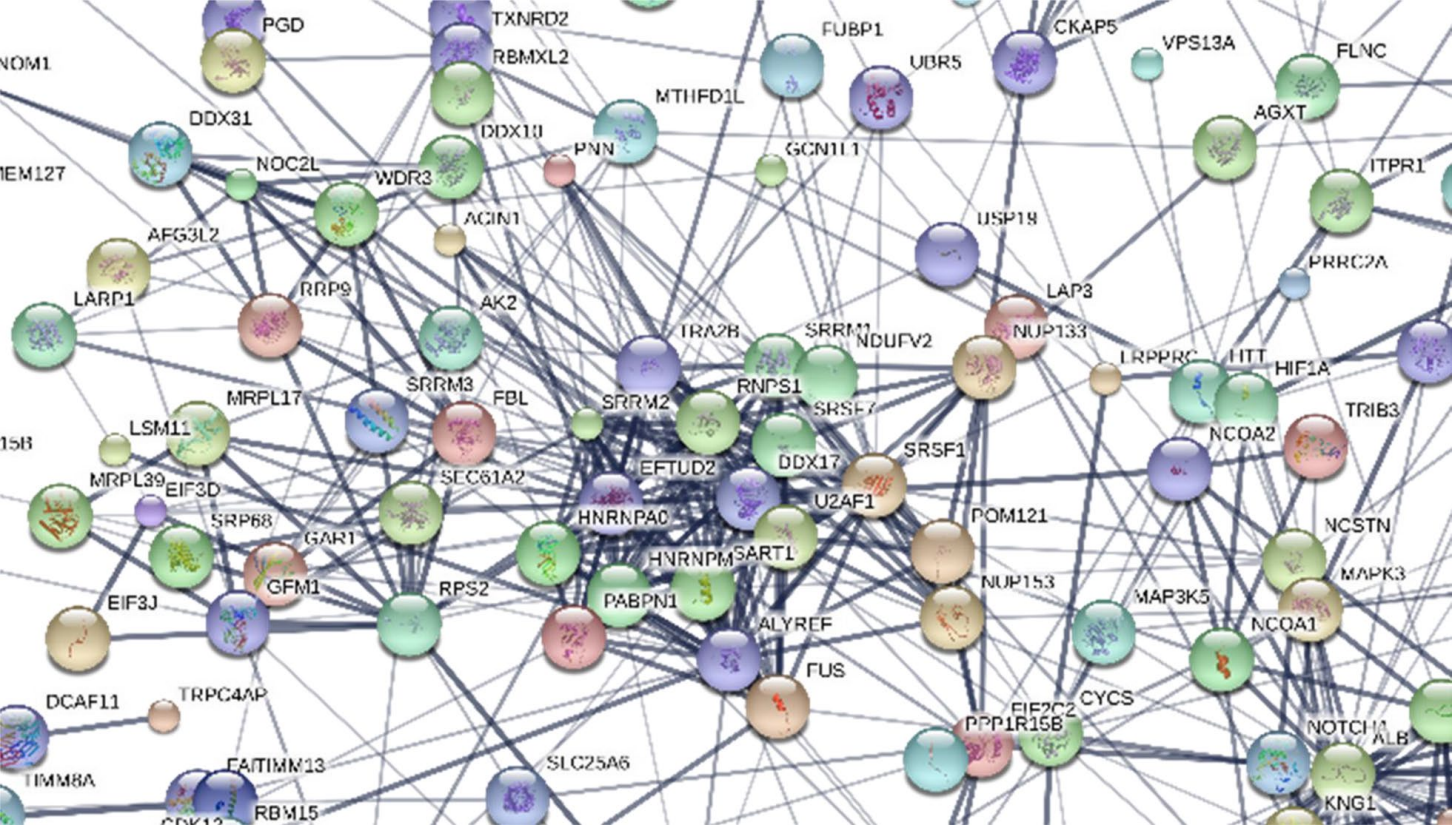
STRING analysis of the top 2000 gene symbols from the endogenous peptides of normal human plasma. Network Statistics: number of nodes: 478; number of edges: 889; average node degree: 3.72; avg. local clustering coefficient: 0.415; expected number of edges, 654; PPI enrichment p value, ≪ 0.0001.  The image shown was cropped from the entire network for the purpose of graphical clarity

In a computationally independent method, the distribution of the known protein–protein interactions, cellular location, molecular function and biological processes of the proteins identified from endogenous peptides were computed with respect to a random sampling of the human genome (Table 3). STRING analysis of the top 2000 gene symbols showed a very tight association of mostly cellular proteins that were apparently present in the plasma as protein complexes [54] with related cellular components, molecular functions and biological processes that strongly support the validity of the SEQUEST algorithm after accepting the best charge state and peptide sequence. Examining the top 600 gene symbols clearly indicated there were many protein interactions apparent between the proteins computed (Fig. 8). The peptidome showed statistically significant enrichment of protein interactions (PPI enrichment p-value ~ 0) and Gene Ontology terms that were consistent with structural and functional relationships between the proteins identified compared to a random sampling of the human genome. The molecular function of the proteins identified by the best fit peptides showed a significant enrichment in proteins that bind macromolecules such as nucleic acids, other proteins and the extracellular matrix to form supramolecular complexes (Table 3). In agreement with previous results that indicated cellular protein complexes may persist in circulation  [54], two of the most commonly observed cellular proteins were RAB21 and DENND5A (DENN/MADD Domain Containing 5A) that is a known RAB binding protein (Fig. 8).

**Table 3:** STRING analysis of the molecular function of proteins identified from endogenous tryptic peptides with respect to a random sampling of the human Genome [60]

Molecular function (GO)			
Pathway ID	Pathway description	Count in gene set	False discovery rate
GO:0044822	Poly(A) RNA binding	64	1.18E−07
GO:0003676	Nucleic acid binding	135	1.77E−06
GO:0003723	RNA binding	72	2.23E−06
GO:0044877	Macromolecular complex binding	55	7.04E−06
GO:0005515	Protein binding	150	3.11E−05
GO:0005198	Structural molecule activity	33	0.000551
GO:0048407	Platelet-derived growth factor binding	5	0.00165
GO:0005488	Binding	271	0.00186
GO:0097159	Organic cyclic compound binding	165	0.00187
GO:0008092	Cytoskeletal protein binding	28	0.00433
GO:1901363	Heterocyclic compound binding	161	0.00433
GO:0032403	Protein complex binding	29	0.0045
GO:0070742	C2H2 zinc finger domain binding	4	0.027
GO:0003682	Chromatin binding	25	0.0276
GO:0005201	Extracellular matrix structural constituent	8	0.0323

## Discussion

About ~ 14,000 (X!TANDEM) to ~ 26,000 (SEQUEST) human proteins (gene symbols, loci and predicted proteins) including many known cellular proteins and protein complexes were confidently detected from the plasma after accepting the distinct, Rank 1 best fit of the MS/MS spectra from the stepwise organic extraction of human EDTA plasma. The large number of proteins detected showed good agreement with previous estimates of the blood proteins by independent methods [14, 18, 54].

### Discovery of endogenous peptides by organic extraction and LC–ESI–MS/MS

Exploiting the apparent tendency of circulating proteases to cleave proteins secreted or otherwise released from cells may be a simple strategy to indirectly discover the cellular proteins in human plasma. The random and independent sampling of the endogenous peptides that were extracted by a step gradient of organic/water seems to have identified most human proteins types from EDTA plasma with at least 5 peptides using SEQUEST [55]. More than ~ 14,000 protein gene symbols were detected with at least 5 peptides using the stringent X!TANDEM algorithm [42] that show low computed FDR (q ≤ 0.0001) by the standard statistical method of Benjamini and Hochberg [56] with the generic R statistical analysis system.

### Random and independent sampling of blood peptides

Random and independent sampling of a population is a standard statistical practice for discovery research. The random sampling of blood peptides from EDTA plasma on separate HPLC columns may be a good practice for discovery of peptides from clinical trials. However, the random and independent sampling of endogenous tryptic peptides generates large amounts of MS/MS data that must be fit, stored, related, filtered, computed, transformed, plotted, and statistically analyzed. The SEQUEST and X!TANDEM algorithms rely on the fit of the MS/MS spectra to the predicted fragments of the human tryptic peptides. The advent of powerful 64-bit PC computing effectively addressed the computational problem that was the limitation on the application of proteomics to compare large populations [57]. The SQL Server/R system may be used to select only the best charge state and peptide sequence for each MS/MS spectra, to compute the cumulative p value and FDR q-values of the results per gene symbol and provide graphical and statistical analysis. The results of LC–ESI–MS/MS once stored in a generic relational database such as SQL Server may then be statistically analyzed at the level of disease or normal control treatments. The SQL Server and open source R data storage and analysis system provides maximal data size compression, and share simple to use, menu-driven or natural language (Boolean Operator) commands. The random and independent sampling strategy together with analysis in SQL Server/R thus permits the comparison of any peptide or protein across different plasma treatments from multiple institutions.

### Sensitivity of LC–ESI–MS/MS for plasma peptides

In theory all proteins should be detectable in human plasma [58] and in this exhaustive experiment about ~ 14,000 human gene symbols, proteins or loci were confidently detected by the X!TANDEM algorithm and about 89% of all known human proteins were detected by the SEQUEST algorithm. The most commonly detected proteins from endogenous tryptic peptides show good agreement with those from exogenous digestion [8, 14, 17, 28]. However, much greater levels of sensitivity for cellular proteins was achieved by the combination of progressive organic extraction together with nanoelectrospray ionization with the linear quadrupole ion trap [41]. The nano LC–ESI–MS/MS system may show sensitivity from micromole to attomole on column [59] and while the instrument is sensitive to any one of these amounts separately  it is difficult to identify peptides at widely different concentrations at the same instant. The purpose of the stepwise organic extraction followed by analytical C18 chromatography was to achieve sufficient separation to ensure that low abundance peptides have the opportunity to ionize without competition or suppression from co-eluting analytes. For a purified protein the limit of automatic identification by LC–ESI–MS/MS is ~ 1 femtomole to 100 attomole on column and so from 0.2 ml of plasma, proteins as low a nanomolar (E-9) and perhaps picomolar (E-12) concentrations may have been detected. Multiple lines of evidence all agree that many, but not all, human proteins were apparently detected and quantified from EDTA plasma by stepwise organic extraction.

### Specificity of LC–ESI–MS/MS for plasma peptides

The protein p-values and FDR corrected q-values computed in R showed that at least 14,000 proteins were confidently detected in human plasma from precursors of ≥ E4 counts that showed low type I error rates in the assignment of molecular identity. The low error rate observed here is consistent with the low error rate of plasma peptides by electrospray compared to random MS/MS and the agreement on plasma proteins from entirely free “no enzyme” computation versus highly constrained fully tryptic peptides [27, 39, 40].

### Selectivity for cellular proteins

The representation of cellular proteins by endogenous tryptic peptides may reflect the stability of the proteins in blood fluids rather than the concentration of the protein in plasma. The organic extraction method showed a skewed preference for cellular proteins compared to the well-known proteins such as albumin, immunoglobulins, apolipoproteins, protease inhibitors and others that might be more resistant to attack from circulating proteases. The apparent selectivity for cellular proteins is a major advantage for discovery via the low molecular weight peptides of human EDTA plasma. The analysis of the tryptic peptides from selective organic extraction of the acetonitrile-insoluble pellet was an efficient means to enumerate the parent cellular proteins from EDTA plasma. It is not clear if the proteins detected reflect their concentration, susceptibility to cleavage by endoproteases, resistance to turn over by exopeptidase, or the combinations. The observed cellular proteins such as Zn Finger proteins showed good agreement with the results of protein partition chromatography followed by tryptic digestion [8, 15], analysis of peptides by Paul ion trap, and confirmation by Western blot [17].

### Confirmation by STRING analysis

A conceptually and mathematically independent means to confirm the validity of the cellular proteins identified from endogenous tryptic peptides was to search for known structural or functional relationships using the STRING algorithm [60]. If cellular proteins are released into circulation by secretion or exocytosis then the protein–protein interactions that existed in the cells might persist to some extent in plasma. The hypothesis that the cellular proteins observed should still show some structural or functional interactions was tested using the STRING algorithm that estimated the probability that the observed protein–protein interaction could occur by random chance approached zero. In agreement with the results of exogenous digestion [54], it appears that proteins circulate as supramolecular complexes and the complex components may proteolytically degrade together in plasma.

### Agreement with independent studies

Immuno depletion, tryptic digestion and ion exchange separation of peptides followed by C18 LC–ESI–MS/MS with “no enzyme” correlation by SEQUEST identified thousands of protein sequences [10–13] from blood from non-tryptic peptides [61] but only a few hundred proteins from high confidence tryptic peptides [62, 63]. Partition chromatography of intact proteins by DEAE resin followed by tryptic digestion and micro spray LC–ESI–MS/MS showed at least 600 different types of proteins were identified by high confidence fully tryptic cross-correlation scores [8] that were independently confirmed [64]. Twelve different partition chromatography columns in parallel, analyzed by the stringent X!TANDEM algorithm showed 4396 proteins from fully tryptic peptides greater than 1,000 counts using micro spray LC–ESI–MS/MS with a Paul ion trap [15]. Adding up all the proteins discovered from plasma to date from multiple institutions using both SEQUEST and X!TANDEM results in an estimate of about 12,000 proteins but only 3858 of these have three independent peptides [14, 19, 54]. Multiple groups reported accessions from different databases that hindered comparison between independent experimental groups [65] however a comparison of the proteins sequences using SQL and BLAST with the Chi Square test clearly demonstrated high levels of agreement between groups with respect to random chance [14, 18, 54]. The rigorous X!TANDEM algorithm fits MS/MS spectra within ± 0.5 Da and provides a p-value for each MS/MS to peptide fit that may be used to compute the cumulative protein p-value and FDR q-value that should be consistent across experiments. Multiple methods agreed that gene symbols with 3 or more independent best fit peptides of greater than 10,000 intensity counts from a linear quadrupole ion trap with X!TANDEM showed a low type I error rate of protein identification (p < 0.001) [27, 38, 48]. The endogenous tryptic peptides were first identified by MALDI Qq-TOF and LC–ESI–MS/MS with an ion trap from C18 collected peptides [28]. A comparison of blood peptide extraction methods by micro electrospray indicated that precipitating the blood fluid in acetonitrile followed by extraction of the pellet was superior to other methods [19, 37, 47] and identified 510 gene symbols with ≥ 5 independent peptides. The combination of stepwise organic extraction with micro-electrospray resulted in the identification of 3463 Gene Symbols of which 1880 had ≥ 5 independent peptides by X!TANDEM (p ≤ 0.0001). Here the combination of step wise organic extraction of 200 μL of plasma with nano electrospray coupled to a linear quadrupole ion trap resulted in the confident identification and quantification of ~ 14,000 gene symbols by X!TANDEM that is the largest number of blood proteins identified to date and shows that you can monitor the ex vivo proteolysis of most human proteins, including interleukins, from blood.

### Pre-analytical variation

Collecting samples onto ice might prevent the secretion of proteins from blood cells, and prevent the degradation of dissolved proteins by proteases, that may occur ex vivo. The effect of ex vivo proteolysis on the observed endogenous peptides of blood samples is known to be large from the use of acid quench, protease inhibitors or ice to preserve the sample [27–29, 47]. We previously showed that plasma from blood collected into EDTA tubes on ice is stable when freeze dried with low peptide frequency and intensity but starts to degrade when dissolved at room temperature [27, 29, 47]. The frequency and/or intensity of peptide or protein observations increased in samples incubated at room temperature compared to ice cold samples and the two pools shared some peptides and proteins [16, 27, 28, 38, 47]. Differences in the frequency of observation and average precursor intensity values of specific cellular proteins like RAB1 or DENDD5A across the clinical samples compared to the ice cold controls indicates the at least some of the peptides and or proteins observed have been released from cells, or degraded by proteases released or activated, ex vivo. The approach of random and independent sampling of tryptic peptides from defined populations using LC–ESI–MS/MS and classical statistics may have some clinical utility: There was apparently statistically significant variation in the cleavage of endogenous peptides from cellular proteins across the different disease and normal treatments, female samples and ice cold controls.

## Conclusion

Multiple independent best fit peptide correlations, low FDR q-values per gene symbol, partition of variation over peptides and proteins, and significant STRING analysis all agreed that most human proteins were identified in EDTA plasma by stepwise organic fractionation, followed by random and independent sampling of the endogenous tryptic peptides by nano LC–ESI–MS/MS fit by the rigorous X!TANDEM algorithm. The use of a stepwise organic solvent gradient for the selective extraction of tryptic peptides into 10 discrete fractions followed by collection and analysis by C18 reversed phase [27] showed high peptide intensity and signal to noise ratios, resulting in LC–ESI–MS/MS correlations to endogenous tryptic peptides from apparently low abundance cellular proteins in blood plasma. The large amounts of raw and calculated data from thousands of LC–ESI–MS/MS experiments from multiple clinical centers can be efficiently stored and related in SQL SERVER and statistically analyzed using the open source R statistical system. It was apparently possible to fractionate the blood fluids using differential solubility in an organic step gradient [38] together with nanospray LC–ESI–MS/MS to make a comprehensive discovery of the endogenous tryptic peptides and phospho peptides from almost all human proteins from EDTA blood plasma. Many of the proteins observed were either secreted from cells, and/or degraded by proteases that were released or activated ex vivo. The detection of the endogenous tryptic peptides may reflect the concentration of the parent protein and/or the stability of the parent proteins in the presence of circulating proteases and/or peptidases in the EDTA plasma. The method was appropriate for discovery of variation in endogenous plasma peptides from cellular proteins that showed significant differences in observation frequency and/or average intensity across the disease and control plasma treatments.

## Additional files

**Additional file 1.** Supplemental Table I. The tryptic peptidome of human EDTA plasma.

**Additional file 2.** Supplemental Table II.  The interleukins, growth factors, cytokines, chemokines and necrosis factors of human EDTA plasma.

**Additional file 3: Table S3.** ANOVA and Tukey Kramer HSD test.
